# IFN-λ1 in CHO cells: its expression and biological activity

**DOI:** 10.1186/s11658-017-0057-x

**Published:** 2017-12-01

**Authors:** Wu-mei Yuan, Wan-ju Zhang, Fen-lian Ma, Jin-song Li, Qian Zhang, Li-shu Zheng

**Affiliations:** 1 Key Laboratory for Medical Virology, Ministry of Health, National Institute for Viral Disease Control and Prevention, China CDC, 100 Ying-Xin St., Xi-Cheng District, Beijing, 100052 China; 2 Key Laboratory of Xinjiang Ethnic Diseases, Department of Biochemistry, School of Medicine, Shihezi University, Shihezi, Xinjiang 830002 China; 3 Shanghai Public Health Clinical Center, Fudan University, Shanghai, 201508 China

**Keywords:** Recombinant human IFN-λ1(rhIFN-λ1), Flp-in-CHO cell, Protein expression, Purification, Biological activity

## Abstract

**Background:**

Many studies have investigated the characteristics and biological activities of type III interferon (IFN), finding that it has similar features to type I IFN but also unique actions because it is recognized by a different receptor.

**Results:**

A full-length recombinant human IFN-λ1 (rhIFN-λ1) cDNA was cloned into the pDF expression vector and stably expressed in Flp-In-CHO cells. After four purification steps (ammonium sulfate precipitation, SP Sepharose chromatography, Blue Sepharose 6 fast flow affinity chromatography and molecular sieve chromatography), the rhIFN-λ1 had a purity of about 90% and was found to have the predicted biological activities. The anti-viral activity of rhIFN-λ1 was determined as 10^6^ IU/mg using the vesicular stomatitis virus (WISH-VSV) assay system. The anti-proliferation activity of rhIFN-λ1 was measured using the MTS method and the growth inhibition ratio was 57% higher than that for recombinant human IFN-α2b (rhIFN-α2b) when the rhIFN-λ1 concentration was 1000 IU/ml. rhIFN-λ1 had lower natural killer cell cytotoxicity than rhIFN-α2b.

**Conclusion:**

The Flp-In-CHO system is suitable for stably expressing rhIFN-λ1 that possesses the predicted anti-viral, anti-proliferation and natural killer cell cytotoxicity-promoting activities.

## Introduction

In 2003, a new type of interferon (IFN) group, which contains the molecules IFN-λ1, IFN-λ2 and IFN-λ3, was identified by two different research groups [1, 2]. These IFNs belong to the type III IFN family. Because of a similar molecular structure to the IL-10 family, they were also called IL29, IL28A and IL28B, respectively. Type III and type I IFNs have similar biological activities, including anti-viral, anti-proliferative and anti-tumor activities, but they have different receptors: an IFN-αR1:IFN-αR2 heterodimer for type I IFNs and an IFN-λR1:IL10R2 heterodimer for type III IFNs [3]. In contrast to the IFN I receptor subunits and the IL-10R2 chain, which are ubiquitously expressed, the IFN-λR1 chain is primarily expressed by epithelial cells and dendritic cells, restricting the action of type III IFNs to these specific cell types [4, 5].

Many type I IFN-based molecules have been widely used in clinical therapy. Pegylated IFN-α2a was approved by the American Food and Drug Administration (FDA) for hepatitis B treatment [6], but severe side effects led to the discontinuation of long-term therapy. Type III IFN was shown to interact with cells of epithelial origin so it caused fewer myelosuppression or neurological side effects. Clinical research with pegylated IFN-λ1 indicated that IFN-λ1 can induce anti-viral activity comparable to that of IFN-α but without the undesirable side effects [7]. Recently, IFN-λ1 has also been used to treat human papilloma infection [8], asthma [9] and lung cancer [10].

In light of the promising clinical applications of type III IFN, we investigated IFN-λ1 more closely using the CHO cell line, which is commonly used in the production of therapeutic proteins [11]. Products expressed by the CHO system can possess similar glycosylation modification to endogenous proteins and can exhibit desirable biological activities. In this study, we established a CHO system to express rhIFN-λ1 and then purified the product and assessed its various biological activities.

## Materials and methods

### Bacterial strains, plasmids and cell lines

Competent *Escherichia coli* DH5α cells and plasmids pMD-18 T (TaKaRa) and pcDNA5/FRT (pDF) (Life Technologies) were used for cloning and protein expression. Flp-In-CHO cells were purchased from Life Technologies. WISH cells, HeLa cells, K562 cells and vesicular stomatitis virus (VSV) have been described previously [12]. RhIFN-α2b was obtained from Yuan-Ce Corporation (Beijing, China). Trypsin and fetal bovine serum (FBS) were purchased from Gibco.

### Recombinant eukaryotic rhIFN-λ1 vector construction

The rhIFN-λ1 gene was amplified via PCR from a pMD18T-λ1 plasmid template from Invitrogen using the following primers: 5′-gctagcatggctgcagcttggaccgtggtgctggtgac-3′ (sense primer: NheI, recognition site underlined) and 5′-aagcttttatcaggtggactcagggtgggttgacgttc-3′ (antisense primer: HindIII, recognition site underlined). The reaction conditions were 95 °C for 5 min; 30 cycles of 95 °C for 30 s, 56 °C for 30 s, and 72 °C for 60 s; and 72 °C for 10 min. The amplicon was then inserted into pcDNA5/FRT (pDF) using the primer restriction sites. The recombinant plasmid, named pDF-λ1, was identified by digestion with NheI and electrophoresis of the digestion products, and was confirmed via sequencing (performed by the Beijing Tsinke Biotechnology Company). The recombinant plasmid was amplified and purified using an EndoFree Plasmid Maxi Kit (Qiagen).

### DNA transfection and screening for high productivity cells

Flp-In-CHO cells (Life Technologies) were maintained in F12 medium (Gibco) supplemented with 10% heat-inactivated fetal bovine serum (FBS; Gibco) at 37 °C in a humidified incubator containing 5% CO_2_. The cells were transfected with 1 μg plasmid (pDF-λ1:pOG44 was 1:9) using Lipo 2000 (Life Technologies) according to the manufacturer’s protocol.

To confirm expression of the recombinant protein, 48 h after transfection, the cells were fixed with methanol, washed three times in PBS, and then incubated with an anti-IFN-λ1 mouse monoclonal antibody (diluted 1:1000; Abcam) at 37 °C for 1 h. After washing three times with PBS, the cells were incubated with a secondary rabbit monoclonal anti-mouse IgG antibody (KPL) conjugated with FITC (diluted 1:2000) at 37 °C for 1 h. Finally, the cells were washed three times in PBS and observed using fluorescence microscopy. The transfected cells were screened in growth culture medium supplemented with 500 μg/ml hygromycin B (Invitrogen). Single clones were obtained by the limited dilution method in 96-well cell culture plates. The quantity of rhIFN-λ1 was evaluated using an ELISA kit (Abcam).

### Identification of clones stably expressing rhIFN-λ1

Clones were cultured in medium supplemented with 500 μg/ml hygromycin B for ten passages. Genomic DNA extracted with a QIAamp DNA Mini Kit (Qiagen) was amplified via PCR and used to determine cell stability. The rhIFN-λ1 contents of the culture media of the 5th and 10th passages were evaluated using an ELISA kit (Abcam). In addition, rhIFN-λ1 protein was detected in the cell culture media of the 5th and 10th passages via western blot. The primary antibody was a mouse anti-human IFN-λ1 antibody and the secondary antibody was goat anti-mouse IgG-HRP (Zsbio).

### Purification of the rhIFN-λ1 protein

RhIFN-λ1 CHO cell culture medium was harvested via centrifugation at 15,000×g for 30 min at 4 °C. Protein was precipitated from the culture medium for 2 h at room temperature after the addition of 40–85% ammonium sulfate. The precipitate was dissolved in buffer A (50 mM phosphate buffer, pH 7.0, 5 mM EDTA) and dialyzed overnight at 4 °C.

The first step was cation exchange chromatography. An SP Sepharose Fast Flow column was equilibrated with 5–10 column volumes of buffer A before the addition of the sample at a rate of 1 ml/min. The column was washed with buffer A to elute the non-bound protein until the absorbance was near zero, which indicated no protein in the eluate. Then buffer B (buffer A containing 0 to 1 M NaCl) was used as a linear gradient to elute rhIFN-λ1 from the column. The elution peak was determined from the rising, plateau and falling absorption values.

The fractions were gathered in different tubes and concentrated using a centrifugal concentrator (Merck). The target protein in the purified samples was then identified using SDS-PAGE and western blot.

A Blue Sepharose 6 Fast Flow affinity column was equilibrated with 5–10 column volumes (column volume was 25 ml) of start buffer (buffer A containing 0.15 M NaCl). Purified sample containing rhIFN-λ1 was added at a rate of 1 ml/min. Unbound protein was washed off the column using start buffer. Then buffer A containing 2 M NaCl (elution buffer A) was used to obtain elution peak 1, and the column was eluted with buffer A containing 50% ethylene glycol to obtain elution peak 2.

Proteins from both elution peaks were condensed via ultrafiltration and assessed using SDS-PAGE and western blot. The rhIFN-λ1 solution was equilibrated with buffer A and finally molecular sieve chromatography was performed using Sephacryl S-100 gel equilibrated with buffer A. After adding the sample at a rate of 0.5 ml/min, the protein was collected in a different tube and concentrated. Because of the different sized molecules in the eluate, fractions with different absorption peaks were collected separately. The various fractions of the elution were assessed using SDS-PAGE and western blot.

### Anti-viral assay for rhIFN-λ1 in WISH cells

WISH cells were seeded in Dulbecco’s modified Eagle medium (DMEM) supplemented with 10% FBS at a density of 10^4^ cells/well in 96-well plates and left overnight. The cells were incubated with successive 4^0^- to 4^9^-fold dilutions of rhIFN-λ1, commercially available rhIFN-λ1 (R&D), or 1000 U/ml rhIFN-α2b (as the standard) for 24 h before being challenged with VSV at the 100 tissue culture infective dose (TCID_50_) in each well.

Cytopathic effects were observed after incubation with the virus for 16–20 h. Crystal violet was added to the wells when all the control cells were dead and the absorbance at 570 nm was measured to determine the degree of cytopathy in different wells.

The anti-viral activity of rhIFN-λ1 was calculated according to the interferon standard, which was defined as one unit of activity when 50% of cells were dead. Using the same method, we detected the anti-viral activity of rhIFN-α2b, rhIFN-λ1, and rhIFN-λ1 (R&D) in A549 cells challenged with VSV.

### Assay for anti-influenza virus activity of rhIFN-λ1

RhIFN-α2b and rhIFN-λ1 were successively diluted to 4^0^- to 4^9^-fold dilutions and incubated with A549 cells for about 16 h. Then anti-influenza virus activity was measured after infection with 100TCID_50_ of influenza virus (A/PR/8/34, H1N1) for 16–20 h, as described above, using crystal violet to measure the degree of cytopathy.

### Determination of anti-proliferation activity of rhIFN-λ1

Anti-proliferation activity was determined using a CellTiter 96 Aqueous One Solution Cell Proliferation Assay kit (Promega) according to the manufacturer’s instructions. HeLa cells were seeded in 96-well plates (5 × 10^4^ cells/well) and then cultured in DMEM containing 10% FBS. After 6 h, the culture medium was discarded and a dilution series of rhIFN-λ1 or rhIFN-α2b was added. After incubation for 72 h at 37 °C, 20 μl of One Solution Reagent was added to each well and incubated for 4 h. The optical density was then measured at A_490_.

### Determination of rhIFN-λ1 activity to promote natural killer cell cytotoxicity

Natural killer cell (NK) cytotoxicity was determined using the dehydrogenase releasing method. Normal human peripheral blood lymphocytes were adjusted to 10^6^ cells/ml using DMEM containing 10% FBS. The NK cells were mixed with K562 target cells at a ratio of 100:1 and then treated with a dilution series of rhIFN-λ1 or rhIFN-α2b for 4 h at 37 °C. The supernatant was then obtained by centrifugation for 10 min at 3000×g. Lactate dehydrogenase content in the supernatant, which represents NK cytotoxicity activity, was measured with the CytoTox 96 Non-Radioactive Cytotoxicity Assay Kit (Promega).

## Results

### Construction of the rhIFN-λ1 expression vector

**Fig. 1:**
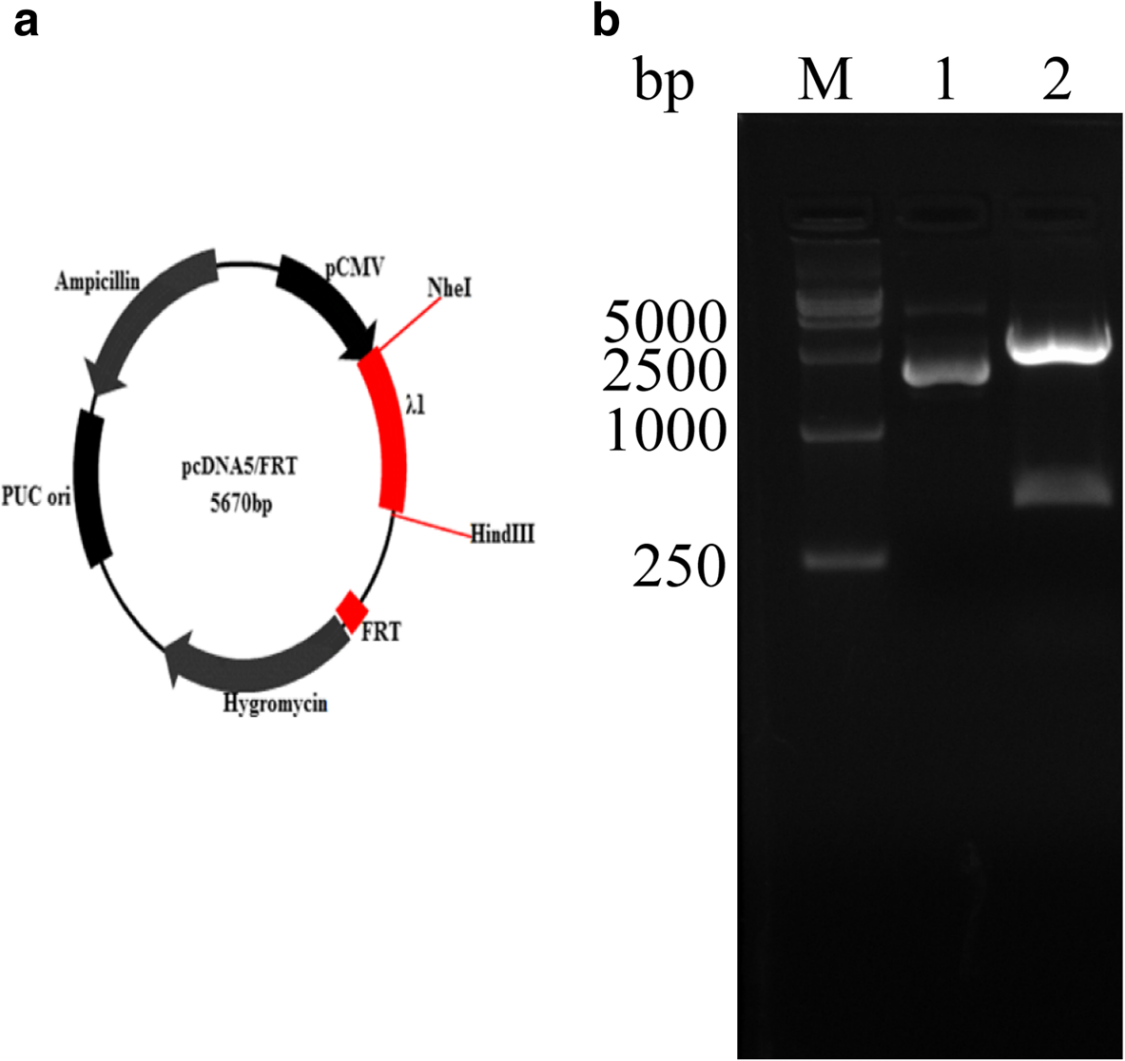
Construction and restriction endonuclease digestion of recombinant pDF-λ1. **a** – Design of recombinant pDF-λ1. **b** – Restriction endonuclease digestion of recombinant pDF-λ1. Lane 1 shows the recombinant vector before digestion. Lane 2 shows the recombinant vector digested by NheI and HindIII. M is the DNA ladder

A full-length 603-bp IFN-λ1 gene sequence was synthesized. The target sequence was ligated into the cloning vector, pMD18T, and then transformed into DH5α cells. The fragment was sequenced to confirm the absence of amplification errors and inserted into the expression vector, pDF (Fig. 1a). The recombinant plasmid, pDF-IFN-λ1, was identified using restriction digestion with NheI and HindIII (Fig. 1b) and sequencing. The recombinant plasmid was amplified and purified, and its concentration was measured for subsequent transfection.

### Identification of rhIFN-λ1-expressing cells

**Fig. 2:**
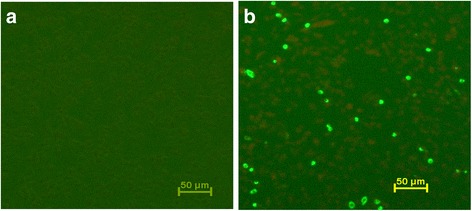
Immunofluorescence 48 h after transfection of Flp-In-CHO cells. **a** – Control group transfected with pDF. **b** – The expression of IFN-λ1 can be detected in cells transfected with pDF-λ1

The expression of rhIFN-λ1 in Flp-In-CHO cells was detected using indirect immunofluorescence (Fig. 2) 48 h after transfection. Fourteen CHO cell clones were obtained that continuously expressed rhIFN-λ1. The concentration of the expressed protein in the culture medium was 3 μg/ml as measured using ELISA. Because only one copy of the target gene was inserted into the genome of the CHO cells, the quantity of rhIFN-λ1 protein was nearly the same in all clones.

### Identification of stable recombinant cell clones

Cell clones were cultured in medium supplemented with 500 μg/ml hygromycin B for ten passages. The rhIFN-λ1 sequence was detected in the 10th passage cells using PCR. The target protein was detected in cell culture supernatants from the 5th and 10th passages using western blot analysis. The protein concentration was still found to be 3 μg/ml using ELISA.

These results show that the rhIFN-λ1 CHO clones were heritably stable. Thus, we obtained a stable CHO cell line that expressed the rhIFN-λ1 protein. Next, we expanded the cell clone and collected the culture medium from protein purification.

### Purification and identification of rhIFN-λ1 protein

**Fig. 3:**
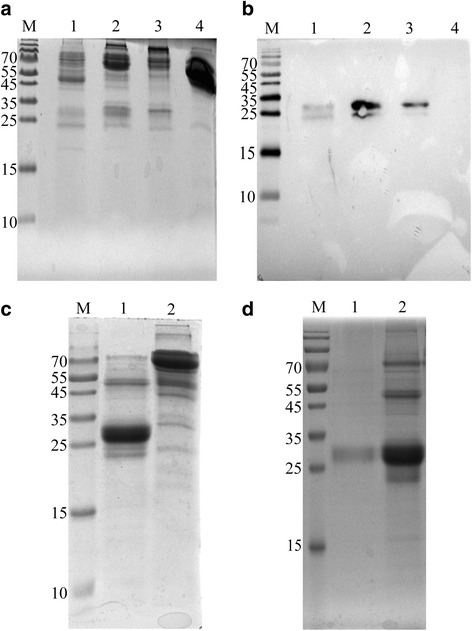
SDS-PAGE and western blot analysis of IFN-λ1. **a** – Cation exchange chromatography elution profile. Lanes 1, 2, and 3 correspond to the three phases of the elution peak: rise, plateau and fall. Lane 4 shows the unbound protein. **b** – The corresponding western blot of the gel in A. **c** – Blue Sepharose 6 Fast Flow affinity chromatography purification results. Lane 1 shows the target protein eluted by buffer A, which containing 50% ethylene glycol. Lane 2 shows the protein in elution peak 1. **d** – Sephacryl S-100 gel purification results. Lane 1 shows the purified IFN-λ1 and lane 2 shows the solution before purification. All the samples were separated using 12% SDS-PAGE and visualized using Coomassie blue staining. M: protein molecular weight marker

RhIFN-λ1 protein was isolated using the classic ammonium sulfate precipitation method as a first step. Most of the acidic proteins were removed using cation exchange chromatography (Fig. 3a and b). Further purification was performed with Blue Sepharose 6 Fast Flow affinity chromatography. A purity of up to 58% was achieved because the gel has a special affinity for interferon and certain other enzymes (Fig. 3c). rhIFN-λ1 purified to 90% was achieved through size exclusion chromatography (Fig. 3d). The isoelectric point of rhIFN-λ1 was 8.1, so cation exchange chromatography was used to purify the target protein. Blue Sepharose 6 has a high affinity for interferon, enabling most impurities to be removed and the final purification by size exclusion chromatography yielded a highly purified rhIFN-λ1.

### Biological activities of rhIFN-λ1

To determine the biological activities of rhIFN-λ1, WISH cells were incubated with a dilution series of rhIFN-λ1 and then attacked with VSV. The number of live cells, shown by staining with crystal violet, reflected the activity of rhIFN-λ1. The results indicated that the anti-viral activity of the purified rhIFN-λ1 expressed by the CHO cells was 10^6^ IU/mg, which was higher than that of the rhIFN-λ1 sold by the company (R&D). The anti-influenza virus activity was up to 3.9 × 10^6^ IU/mg in A549 cells, which was markedly lower than that of rhIFN-α2b (Table 1).

**Table 1:** Comparison of the anti-viral activity of rhIFNs in different cell lines

Interferons	Biological activity (IU/mg)		
	WISH VSV	A549 VSV	A549 A/PR/8/34
rhIFNα-2b	1.2 × 10^8^	1.8 × 10^8^	1.8 × 10^8^
rhIFN-λ1	1.06 × 10^6^	1.4 × 10^6^	3.9 × 10^6^
rhIFN-λ1 (R&D)	1.1 × 10^5^	1.2 × 10^6^	2.0 × 10^6^

**Fig. 4:**
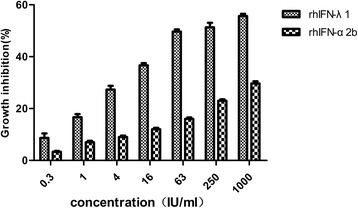
Comparison of anti-proliferation activity between rhIFN-λ1 and rhIFN-α2b. The anti-proliferation activity of rhIFN-λ1 and rhIFN-α2b on HeLa cells was detected using the MTS method

**Fig. 5:**
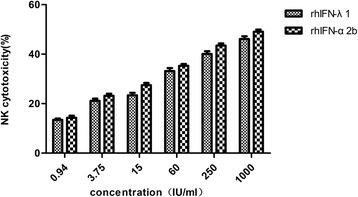
Comparison of NK cytotoxicity promotion between rhIFN-λ1 and rhIFN-α2b. NK cytotoxicity was determined via the dehydrogenase releasing method using the Cytotox 96 Non-Radioactive Cytotoxicity Assay Kit. The ratio of NK cells to K562 target cells was 100:1

A comparison of anti-proliferation activity between rhIFN-λ1 and rhIFN-α2b using the MTS method showed that both IFNs had dose-dependent growth suppression activity. The growth inhibition ratio of rhIFN-λ1 reached 57% at a concentration of 1000 IU/ml, which was higher than that of rhIFN-α2b (Fig. 4). rhIFN-λ1 promotion of NK cytotoxicity increased with dose and was lower than that of rhIFN-α2b (Fig. 5).

The reason for the high activity of our purified rhIFN-λ1 may be that our protein had no tag and was expressed from CHO cells. Even though the anti-viral and NK cytotoxicity-promoting activities of rhIFN-λ1 were lower than those of rhIFN-α2b, its biological activity was good and it shows promise for clinical application.

## Discussion

Recent studies have shown that type III interferons (IFNs) possess similar biological activities to type I IFNs. IFN-λ can inhibit hepatitis B replication in human cells [13] and has a similar effect to IFN-α in hepatitis C virus (HCV) patients [14]. Type III IFN can inhibit many other types of virus, including herpes simplex virus (HSV) [15] and cytomegalovirus (CMV) [16], similarly to type I IFN. Type III IFN also participates in the acquired immune response [17] and can inhibit cancer cells with potential for clinical application [15].

IFN-λR1, which is a type III IFN that is strictly expressed by certain kinds of epithelial cells and specific subsets of immune cells, has a different receptor subunit to its type I IFN counterparts [18]. This means that IFN-λ therapy may cause fewer side effects than IFN-α therapy, which usually causes intolerable side effects. An HCV therapy study indicated that pegylated IFN-λ1 possessed similar anti-viral activity to pegylated IFN-α2b but with fewer side effects [19]. Furthermore, the characteristic of tissue-restricted expression of the IFN-λ receptor makes IFN-λ1 a promising candidate for clinical application, especially for some diseases of epithelial tissue in the respiratory, gastro-intestinal and reproductive tracts. For example, there is evidence that the type III IFN system plays an important role in the treatment of asthma [20].

Our expression system in CHO cells expressed rhIFN-λ1 stably and continuously. Flp-In-CHO cells contain the same chromosomal FRT site as the vector, so when the recombinant pDF-IFN-λ1 and helper plasmid pOG44 are co-transfected into Flp-In-CHO cells, the flp recombinase encoded by pOG44 can mediate homologous recombination between the exogenous gene and host DNA, resulting in stable expression. Here, ammonium sulfate precipitation and three chromatography steps were used to purify the target protein because rhIFN-λ1 was not fused to a tag for purification. This is because recombinant protein drugs for clinical use should not possess a tag. The purified protein possessed high anti-viral activity. Furthermore, the CHO expression system provides similar post-translational glycosylation to that found on the endogenous human protein [21]. These results lay the foundation for developing an rhIFN-λ1 that can be clinically applied.

## Conclusion

We obtained stable CHO cell clones expressing rhIFN-λ1. The purified protein possessed high anti-viral activity. Future studies will use the rhIFN-λ1 described here to investigate functional mechanisms and biological applications of rhIFN-λ1.

## Abbreviations

CMV: Cytomegalovirus
DMEM: Dulbecco’s modified Eagle medium
FBS: Fetal bovine serum
FDA: Food and Drug Administration
HCV: Hepatitis C virus
HSV: Herpes simplex virus
IFN: Interferon
IL-10: Interleukin-10
NK: Natural killer cell
pDF: pcDNA5/FRT
rhIFN-λ1: Recombinant human IFN-λ1
TCID_50_: Tissue culture infective dose
WISH-VSV: WISH-vesicular stomatitis virus
